# Dual- or single rinse? The tubular sealer penetration of endodontic chelating agents

**DOI:** 10.1371/journal.pone.0303377

**Published:** 2024-06-14

**Authors:** Beliz Ozel, Tuba Ayhan, Figen Kaptan, Fikrettin Sahin, Meriç Karapınar-Kazandağ

**Affiliations:** 1 Department of Endodontics, Yeditepe University Faculty of Dentistry, Yeditepe University, Istanbul, Turkey; 2 Department of Genetics and Bioengineering, Faculty of Engineering, Yeditepe University, Istanbul, Turkey; Nair Hospital Dental College, INDIA

## Abstract

**Introduction:**

In this study, we aimed to compare the effectiveness of various chelating agents, ethilenediaminetetraacetic acid (EDTA), citric acid (CA), and etidronic acid (HEDP) mixed in two different forms, in removing the smear layer and promoting the penetration of an endodontic sealer into the dentinal tubules of extracted single-rooted teeth.

**Methods:**

The study used 75 teeth divided into five groups: 17% EDTA, 10% CA, 9% HEDP + NaOCl, 9% HEDP + distilled water (DW), and a control (DW) group. Scanning electron microscopy was used to assess smear layer removal and confocal laser microscopy was used to evaluate tubular sealer penetration at different depths from the apical tip.

**Results:**

Sealer penetration was highest with 17% EDTA and 10% CA as compared with the other agents (*p*<0.001). At the cervical third, the sealer penetration for EDTA, HEDP + NaOCl, and HEDP + DW groups were significantly different than those in DW (*p* **=** 0.020). For the middle third, EDTA, CA, and HEDP + NaOCl groups were significantly higher than those of the DW group (*p***<**0.001). Cervical-level values were significantly higher than apical-level values for HEDP + NaOCl, HEDP + DW, and DW (*p***<**0.001). Smear layer removal was lower with 9% HEDP + DW than with 17% EDTA and 10% CA at all depths (*p*<0.001). A significancy in smear layer removal was observed between 10% CA and control (*p* = 0.015) in middle depth.

**Conclusion:**

Within the limitations of this study, highest values were seen in EDTA and CA in terms of sealer penetration and smear layer removal. In the light of these findings, the use of strong chelating agents highlights better clinical efficiency than dual-rinse or single HEDP irrigation.

## Introduction

Teeth that need root canal treatment require inhibition or elimination of microbial infection inside the root canal system [1]. The efficacy of root canal treatment depends on chemomechanical preparation, fluid-tight sealing of the root canal through obturation, and deep sealer penetration [2]. However, contact of a metallic endodontic instrument with the root canal wall forms a smear layer that obstructs the dentinal tubules opening, thus, preventing proper sealing of the root canal system [3].

The smear layer prevents root canal irrigation, medication use, and sealant penetration. Therefore, current recommendations advocate for the removal of the smear layer before obturation [4]. Smear layer removal improves the adaptation of the root-filling material to dentin [3]. Although the smear layer has been extensively studied, its removal method is unknown.

To overcome this problem, clinicians must understand the smear layer properties. A 0.5–2-μm smear layer can cover the dentinal wall and penetrate approximately 40 μm into the dentinal tubules, containing inorganic debris and organic components, including pulpal remnants, bacteria, and blood cells [3, 5, 6]. The irrigant used for smear layer removal can significantly affect the elimination of the inorganic dentin matrix, allowing disinfectants to enter the dentinal tubules.

Smear layer removal can be improved using various irrigants and solution delivery methods [7]. Sodium hypochlorite (NaOCl) is an essential endodontic irrigant that disintegrates organic tissues and acts as an antibacterial agent. However, this did not affect the inorganic content [8]. Therefore, final irrigation with a demineralizing agent, such as ethylenediaminetetraacetic acid (EDTA) or citric acid (CA), improves the dentinal wall permeability by removing the smear layer [9, 10]. CA and EDTA efficiently removed smear layers at similar concentrations [11]. However, EDTA and CA interact with NaOCl [12] to rapidly reduce the available chlorine in the solution, rendering the irrigant ineffective for organic tissues [11]. Owing to their highly acidic environment, they decrease calcium ions in the dentin complex, alter the dentinal matrix, and decrease dentin flexural strength and durability [13]. NaOCl and EDTA degrade dentin by targeting the organic and inorganic components [14]. Therefore, a final irrigant that minimally compromises the dentinal structure and efficiently removes the smear layer to improve sealer penetration into the dentin would be beneficial in a clinical setting.

Various final irrigants are being frequently used in endodontic clinical practice. One of these, EDTA, has gained wide popularity due to its strong ability to form a ‘sequester’ of Ca2+ and Fe3+ which arises the chelating capacity of the solution. The normal concentration in clinical practice is 17% where the solution removes smear layer less than a minute after being in contact with the root canal surface [15]. Similarly, citric acid, as a tricarboxylic acid, removes the smear layer through detoxifying the root surface and therefore, exposing the collagen fibres [16]. Even though both solutions were compared frequently within each other, EDTA remains as the gold standard [13].

Etidronic acid or 1-hydroxyethane-1,1-diphosphonic acid (HEDP) has gained popularity because of its chelating effect, which minimally interferes with the action of NaOCl [17]. HEDP has a unique short-term compatibility (2–4 h) with NaOCl, allowing the application of a combined NaOCl/sHEDP solution during chemomechanical preparation or final irrigation while maintaining the proteolytic/antibacterial effects of NaOCl [17, 18]. The combined use of HEDP with NaOCl, also called dual-rinse, leads to “continuous chelation,” a beneficial outcome of their short-term compatibility. This continuous chelating effect minimizes dentinal structure destruction and considered as ‘less-toxic’ [19].

Additionally, HEDP prevents the formation of smear layers and debris [20]. When mixed with distilled water, HEDP alone can be used as a chelating agent in clinical setting [21]. It functions as a single irrigant in the absence of NaOCl. Still, the use of HEDP is advised to be in combination with NaOCl. It remains unknown whether this combination acts in favor of the ‘tissue-dissolving’ effect of NaOCl [15] and HEDP alone, as the final irrigant, improves smear layer removal and tubular sealer penetration.

A long-term investigation of the efficacy of chelating agents based on the sealer penetration depth and percentage determined using confocal microscopy has been reported [22]. Despite the lack of clinical relevance [23], sealer penetration depth is still used to evaluate irrigation or obturation systems [22, 24, 25]. SEM is the primary tool used for root canal sealing, smear layer removal, and tubular dentin penetration [10, 26]. Its limitations include sample polishing and coating, low beam penetration depth, voltage contrast, and artifacts observed in images [27, 28]. In contrast, CLSM provides high-resolution optical sections of thin, semitransparent samples and images of the surfaces, similar to the SEM method but without issues related to specimen preparation [29]. Because of these superior properties, CLSM has been extensively used to evaluate tubular sealer penetration [30, 31], root dentin alterations [32, 33], and bacterial elimination from dentinal tubules [34, 35]. Table 1 summarizes relevant literature.

**Table 1:** Literature review.

					Protocol		Evaluation		
Year	Author	Journal	MAF	Specimen	Final Irrigation	Intervention	Method	Criteria	Outcome
1994	Silberman	Lasers in Surg and Med	-	Dentin specimen n = 18	-	10% maleic acid+ CO_2_ laser activation	SEM	Smear layer removal	CO_2_ laser increases smear layer resistance
1996	Love	Int End J	-	Palatal root cut in half n = 2	-	Tetracycline affected dentine	SEM+CSLM	Dentin mineralization appearance	Dentine affected by systemic tetracycline
1998	Kimura	Lasers in the Life Sciences	-	sectioned dentin slices n = 30	-	CO_2_ laser irradiation	CSLM + SEM	Dentin ablation	CSLM gave better images in deeper areas
2004	Kokkas	JOE	#35	root cut in half n = 64	3 mL 17% EDTA + 3 mL 1% NaOCl (1 min)	AH Plus, Apexit, Roth 811	SEM	Max. penetration depth	Presence of smear layer affects sealer penetration
2007	Gharib	JOE	#40	Root dentin slices n = 30	3 mL 17% EDTA + 3 mL 6% NaOCl (1 min)	Resilon + Epiphany	Confocal laser microscopy	Percentage of sealer penetration + max penetration depth	Less penetration in apical third
2010	Moon	JOE	#30	Root dentin slices n = 45	3 mL 17% EDTA + 3 mL 1% NaOCl (1 min)	AH Plus	Confocal laser microscopy	Percentage of sealer penetration + max penetration depth	No effect
2011	Ma	JOE	-	Semicylindirical dentin slices N = 6	5.25% NaOCl + 6% citric acid	*E*. *faecalis*	Confocal laser microscopy	Elimination of *E*. *faecalis*	A new model introduced
2011	Balguerie	JOE	#30	Root dentin slices n = 52	3 mL 15% EDTA 3 min + 5 mL 3% NaOCl	Silicon, calcium-hydroxide resin, zinc oxide eugenol, glass ionomer, epoxy resin	SEM	Sealer adaptation to canal wall	AH Plus optimal for penetration
2012	Kara-Tuncer (33)	JOE	#40	Root dentin slices n = 32	5 mL 17% EDTA + 2.5% 5 mL NaOCl 1 min; 5 mL maleic acid + 2.5% NaOCl 1 min; 10% citric acid + 2.5% NaOCl 1 min; 2.5% NaOCl 1 min	AH26	Confocal laser microscopy	Percentage of sealer penetration+ max. Penetration depth	EDTA, MA and CA affected sealer penetration
2013	Bolles	JOE	-	Root dentin slices n = 50	17% EDTA + 6% NaOCl	EndoActivator, Vibringe, Conventional needle	Confocal laser microscopy	Percentage of sealer penetration	No additional effect
2014	Kuçi	JOE	-	Root dentin slices n = 45	2.5% EDTA 3 min + 2.6% NaOCl 1 min; 2.6% NaOCl 1 min	-	Confocal laser microscopy + SEM	Percentage of sealer penetration + smear layer removal	Effect of smear layer removal
2015	Jardine	Clin Oral Invest	#30	Root dentin slices n = 80	2 mL 1.3% NaOCl + 5 mL of QMix, MTAD or EDTA17% 2 min + 5 mL saline 2 min	-	Confocal laser microscopy + SEM	Percentage of sealer penetration + smear layer removal	Similarity in Qmix and EDTA
2016	Azim	JOE	#25	Root dentin cut in half n =?	2 mL 17% EDTA 1 min + 3 mL 6% NaOCl	EndoActivator, Needle Irrigation, XPShaper, PIPS	Confocal laser microscopy	Elimination of *E*. *faecalis*	XPShaper has the highest effect
2016	mcMichael	JOE	#40	Root dentin slices n = 80	3 mL 17% EDTA 1 min + 3 mL 6% NaOCl 1 min + 5 mL saline 1 min	Single cone vs. continuous wave	Confocal laser microscopy	Percentage of sealer penetration	Similarity in both techniques
2017	Machado	Microscopy Research & Technique	-	Root dentin slices n = 52	17% EDTA 3 min, 10% citric acid 3 min, 2.5% NaOCl 3 min	EndoActivator conventional needle EndoVac ultrasound	Confocal laser microscopy + SEM	Percentage of sealer penetration + smear layer removal	EA and EV had similar results
2017	Jeong	JOE	#40	Root dentin slices n = 100	10 mL 17% EDTA 3 min + 3 mL 5.25% NaOCl 3 min + 10 mL deionized water	GP single cone GP vertical condensation CPoint single cone	Confocal laser microscopy	Percentage of sealer penetration + max penetration depth	No significant difference
2018	El Hachem	Clinical Oral Investigations	#40	Root dentin slices n = 96	10 mL 17% EDTA 3 min + 3 mL 5.25% NaOCl 3 min + 10 mL deionized water	AH Plus BC Sealer Novel tricalcium silicate	Confocal laser microscopy	Percentage of sealer penetration + max. Penetration depth	BC and NTS better than AH Plus
2018	Piai	Microscopy Research & Technique	#40	Root dentin slices n = 20	2 mL 17% EDTA 3 min + saline	AH Plus Sealer Plus	Confocal laser microscopy	Percentage of sealer penetration+ max. Penetration depth	Sealer Plus (similar to AH Plus)
2020	Matos	Nature Scientific Reports	#40	Root dentin slices n = 80	17% EDTA 2 min, QMix 2 min	Conventional irrigation, Passive Ultrasonic Irrigation	Confocal laser microscopy+ SEM	Percentage of sealer penetration + smear layer removal	PUI and Qmix gave improved results
2021	Yılmaz	BioMed Research International	#30	Root dentin slices n = 65	3 mL 17% EDTA + 3 mL 5.25% NaOCl	Manual Dynamic Activation, Sonic Irrigation, Passive Ultrasonic Irrigation, Conventional Needle Irrigation	Confocal laser microscopy	Percentage of sealer penetration + max penetration depth	PU, SI, and MD did not gave significant results
2021	Gawdat	Australian Endodontic Journal	#30	Root dentin slices n = (only from 5 mm to the apex)	5 mL 2.6% NaOCl/9% HEDP 5 mL 2.6% NaOCl 5 mL 17% EDTA	-	Confocal laser microscopy	Percentage of sealer penetration + max penetration depth	9% HEDP + NaOCl showed increased penetration area
2022	Kfir	Clinical Oral Investigations	#30	Roots longitudinally splitted to expose root canal walls	2 mL 17% EDTA (0.5 min) 10 mL 9% HEDP + NaOCl (4–5 min, including preparation and final rinse)	-	SEM	Smear layer removal	9% HEDP + NaOCl showed increased smear layer removal

Existing literature [36, 37] provides promising outcome of dual-rinse irrigation when compared to strong chelators such as EDTA or citric acid, however, it is possible that the used concentration or irrigation sequence of these solutions may not follow the advised clinical protocol. Therefore, it is possible to say that the literature still lacks the knowledge of the use of HEDP in various clinical scenarios. In this study aimed to compare the efficacy of chelating solutions (17% EDTA, 10% CA, and 9% HEDP) used in root canal treatment for smear layer removal and tubular sealer penetration using scanning electron microscopy (SEM) and confocal laser scanning microscopy (CLSM) of specific regions of the root canal. The null hypothesis was that the experimental and control groups would have similar smear-layer removal and sealer penetration rates.

## Materials and methods

The study design was based on studies by Machado et al. [38], Kfir et al. [36], and Gawdat et al. [37] and followed the CONSORT guidelines. Ethical approval was obtained from the Yeditepe University Research Ethics Committee (approval no. 1527). All patients provided a written informed consent form prior to sample collection.

### Sample selection

**Fig 1:**
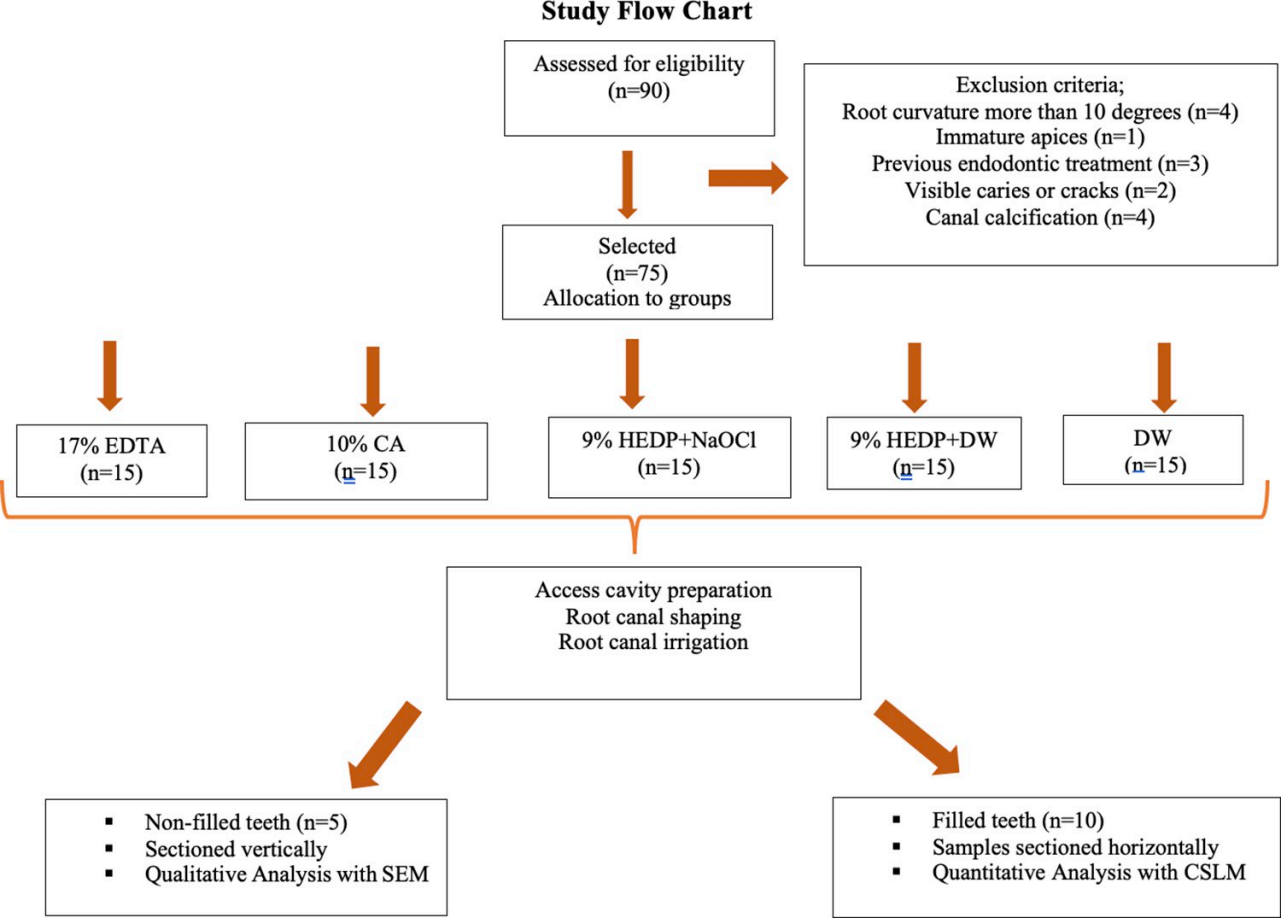
Study flow chart presenting the experimental set-up.

A pilot study (n = 10) according to a previous study [38] was conducted to determine the adequate sample size. The mean penetration percentage and depth differences for 17% EDTA, 10% CA, 9% HEDP + NaOCl, 9% HDP + distilled water (DW), and DW were 8.19% and 1.06 ± 0.11 mm, with d = 0.60 indicating the highest sample size at α = 0.05 and (1-β) = 0.80. Calculations were made with G*Power v 3.1. After obtaining ethical approval, the teeth of patients aged 19–30 years were collected for periodontal extraction. The teeth were stored in 0.1% tylenol at 4°C before use. The calculus and debris were removed, and the defects were examined under a surgical operating microscope (Carl Zeiss, Oberkochen, Germany). These included teeth with straight roots, a root curvature of no more than 10°, as measured according to the methods of Schneider et al. [39], and similar lengths and canal shapes (oval). The canal configuration was confirmed on radiographic images obtained both mesiodistally and buccolingually. The exclusion criteria were presence of caries, immature apices, previous root filling, root resorption, canal calcification, or cracks. After exclusion, 75 teeth were included in this study. Samples, including those used for CLSM (n = 10) and SEM (n = 5) analyses, were allocated to the study groups (n = 15) using a sequence generator (www.random.org) at a 1:1 ratio (Fig 1).

### Study groups

After cavity preparation, root canal length was measured using a #10 K-file (Dentsply Maillefer, Ballaigues, VD, USA) inserted into the canal until it was visible at the apical foramen. A standardized length of 19 mm was obtained for each sample by grinding, and the working length was set to 1 mm short of this length. The apical foramen was sealed with wax.

The root canals were randomly assigned to one of five groups based on the final irrigation protocol.

- Group 1: 17% EDTA (Rehber Kimya, Istanbul, Turkey) (n = 15)
- Group 2:10% CA (*1 g CA + 10 mL distilled water)* (n = 15)
- Group 3:9% HEDP+ NaOCl (Medcem, Darmstadt, Germany) (*0*.*9 g of powder + 10 mL 5*.*25% NaOCl)* (n = 15)
- Group 4:9% HEDP+ DW (Medcem) (*0*.*9 g of powder + 10 mL distilled water) (n = 15)*
- Group 5: DW (Rehber Kimya) (n = 15)

All canals were prepared using ProTaper Next rotary instruments (Dentsply) with an apical diameter of 0.4 mm (#X4) to ensure the instrument touched the canal walls. The canals were irrigated with 5.25% NaOCl (2.5 mL) for 2 min after instrumentation with each file. The same protocol was used for Group 3, except that 9% HEDP + NaOCl irrigation was used throughout the preparation. Irrigation was performed using side-vented irrigation needles adjusted according to the canal length (PPH Cerkamed, Nisko, Poland). All canals were irrigated with 5 mL of the designated final rinse solution for 1 min [24] and activated with an EndoActivator 25/.05 tip (Dentsply Maillefer) for 30 s, followed by irrigation with 5 mL of DW. Finally, the canals were dried using 40 paper points (DiaDent, Seoul, South Korea). All intervensions were done by an experienced endodontist of 10 years (T.A.)

### SEM analysis of smear layer removal

Five unfilled samples from each group were examined using SEM. The sample size was determined using the method described by Machado et al.’s method [38]. According to Ulusoy et al. [40], longitudinal splitting of the root was performed to expose the root canal system and ensure no decontamination of the canal lumen. Then, the samples were subjected to gradual dehydration by immersion in increasing ethanol concentrations (25–100%) and placed in a low-vacuum pressure desiccator for 24 h before imaging [41]. Imaging areas were standardized for all samples by marking distances of 3, 5, and 8 mm from the root apex. The sections were mounted on circular metal stubs and covered with a 30-nm thick layer of gold-platinum. All images were obtained by an independent technician blinded to the study groups through SEM under 15 kV and 25 spot sizes (Tokyo, Japan) using the closed technique. Two examiners performed the calibration by analyzing 12 images using the scoring system proposed by Torabinejad et al. [42]. The interobserver agreement was 83.3% (Fleiss kappa 0.7). Immediately thereafter that, ten images different from those taken for calibration were obtained from each third of the root canals for analysis under different magnifications (1000× and 2000×). Examiners who were blinded to the study group scored the images. The data were analyzed using the Kruskal–Wallis test, followed by the Dunn–Bonferroni post-hoc test, where the significance level was set at 95%.

### CLSM for sealer penetration evaluation

An AH Plus sealer dyed with 0.1% Rhodamine B (Merck, Rahway, NJ, USA) was placed inside the canals using a #25 PastInject (MicroMega, Besancon, France) operated under rotary motion at 600 rpm. All the teeth were obturated using a #40 master gutta-percha (DiaDent) cone followed by lateral condensation. Excess gutta-percha was removed using a GuttaCut (VDW, Munich, Germany). All experimental procedures were performed by the same operator. The teeth were placed in an incubator (Esco Scientific, Singapore) at 37°C and 100% humidity for 24 h to allow the root canal sealer to set (Fig 1). Horizontal sections were obtained using a water-cooled 0.6-mm thick microtome saw (Buehler, Uzwil, Switzerland) at 3, 5, and 8 mm from the root apex to obtain dentin sections. The coronal surfaces of the slices were polished with a silicon abrasive carbide paper to reduce their thickness to 0.5 mm. The apical surfaces of the samples were placed on glass slides and labeled. All sections were then examined using CLSM (Carl Zeiss) with a solid laser, 532-nm resolution, tile scan mode (tile size, 7 × 7 pixels), 0.3 lens aperture, and magnification of approximately 10. The percentage of sealer penetration and the maximum penetration depth were determined by measuring the regions where the sealer penetrated the dentinal tubules along the root canal walls using the ImageJ V 1.53e (US National Institute of Health, Bethesda, MD, USA) measurement tool (https://imagej.nih.gov/ij/), which was then divided by the circumference of the root canal wall and multiplied by 100 to calculate the percentage [28]. Maximum penetration depth was measured by drawing a line from the canal wall to the deepest point where the sealer could be visualized. To calibrate the pixels used, a scale was set by matching a known distance of 1 mm with the distance between the pixels.

### Statistical analysis

Statistical analyses were performed using R ver. 2.15.3 (R Core Team, Vienna, Austria), and the significance level was set at 95% (*p*<0.05). Data are reported as means, standard deviations, medians, and first- and third-quarter values. The Kolmogorov–Smirnov test was used to verify the normality assumption. The non-parametric Kruskal–Wallis test followed by the Dunn–Bonferroni post-hoc test was used to evaluate the mean tubular sealer penetration based on two outcomes: total penetration percentage (%) and maximum penetration depth (mm), and to compare the sealer penetration percentage at each canal depth.

## Results

### Smear layer removal

**Fig 2:**
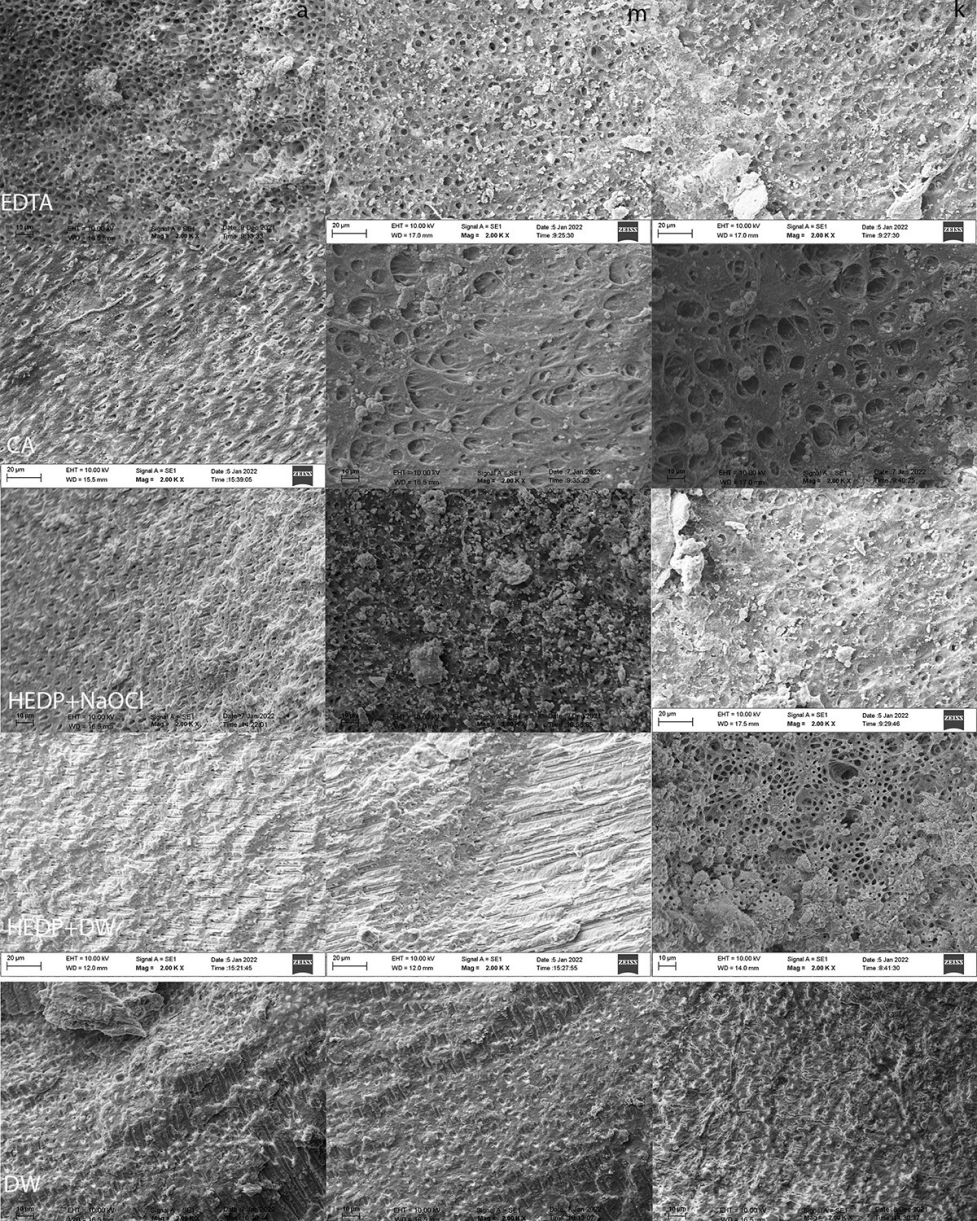
Representative SEM images of each chelating agent group (rows) at each sectional level (columns). Rows representing the chelating agent groups: (1) EDTA, (2) HEDP, (3) CA and (4) DW. Columns representing the root depth; (a) apical, (b) middle, (c) coronal. CA, citric acid; DW, distilled water; EDTA, ethylenediaminetetraacetic acid; HEDP, etidronic acid; SEM, scanning electron microscopy.

Fig 2 shows the representative images of each group at each canal depth. Comparisons between chelating agents considering the root canal depth showed the lowest smear layer removal in HEDP + DW when compared to EDTA, CA, and HEDP + NaOCl groups in apical and cervical depth (*p*<0.001). At the middle level, a significance was observed only between the CA and control groups (*p* = 0.015) (Table 2). HEDP+NaOCl removed less smear layer at the apical depth when compared to cervical and middle levels (*p* = 0.028).

**Table 2:** Smear layer removal at each section level.

Smear layer removal		Apical	Middle	Cervical	*p*-value
**EDTA**	Mean±SD	1.75±0.46^Ba^	1.75±0.46^ABa^	1.38±0.52^Ba^	0.216
	Median (IQR)	2 (1.5–2)	2 (1.5–2)	1 (1–2)	
**CA**	Mean±SD	1.9±0.32^Bb^	1.6±0.52^Bb^	1.7±0.48^ABb^	0.294
	Median (IQR)	2 (2–2)	2 (1–2)	2 (1–2)	
**HEDP + NaOCl**	Mean±SD	2.2±0.42^Bc^	1.8±0.63^ABcd^	1.5±0.53^Bd^	0.028*
	Median (IQR)	2 (2–2)	2 (1–2)	1 (1–2)	
**HEDP + DW**	Mean±SD	2.9±0.32^ABe^	2.3±0.67^ABe^	2.5±0.53^Ae^	0.055
	Median (IQR)	3 (3–3)	2 (2–3)	2 (2–3)	
**DW**	Mean±SD	2.5±0.53^Af^	2.4±0.52^Af^	2.3±0.48^ABf^	0.668
	Median (IQR)	3 (2–3)	2 (2–3)	2 (2–3)	
	***p*-value**	<0.001*	0.015	<0.001*	

The values are presented as means±SD or medians (IQR).

*Kruskal–Wallis test (*p*<0.05). Uppercase superscript letters indicate statistical differences between columns (A-B). Lowercase superscript letters indicate statistical differences between rows (a-f). Dunn–Bonferroni post-hoc test (*p*<0.05).

CA, citric acid; DW, distilled water; EDTA, ethylenediaminetetraacetic acid; HEDP, etidronic acid; IQR, interquartile range; SD, standard deviation

### Sealer penetration percentage and depth

**Fig 3:**
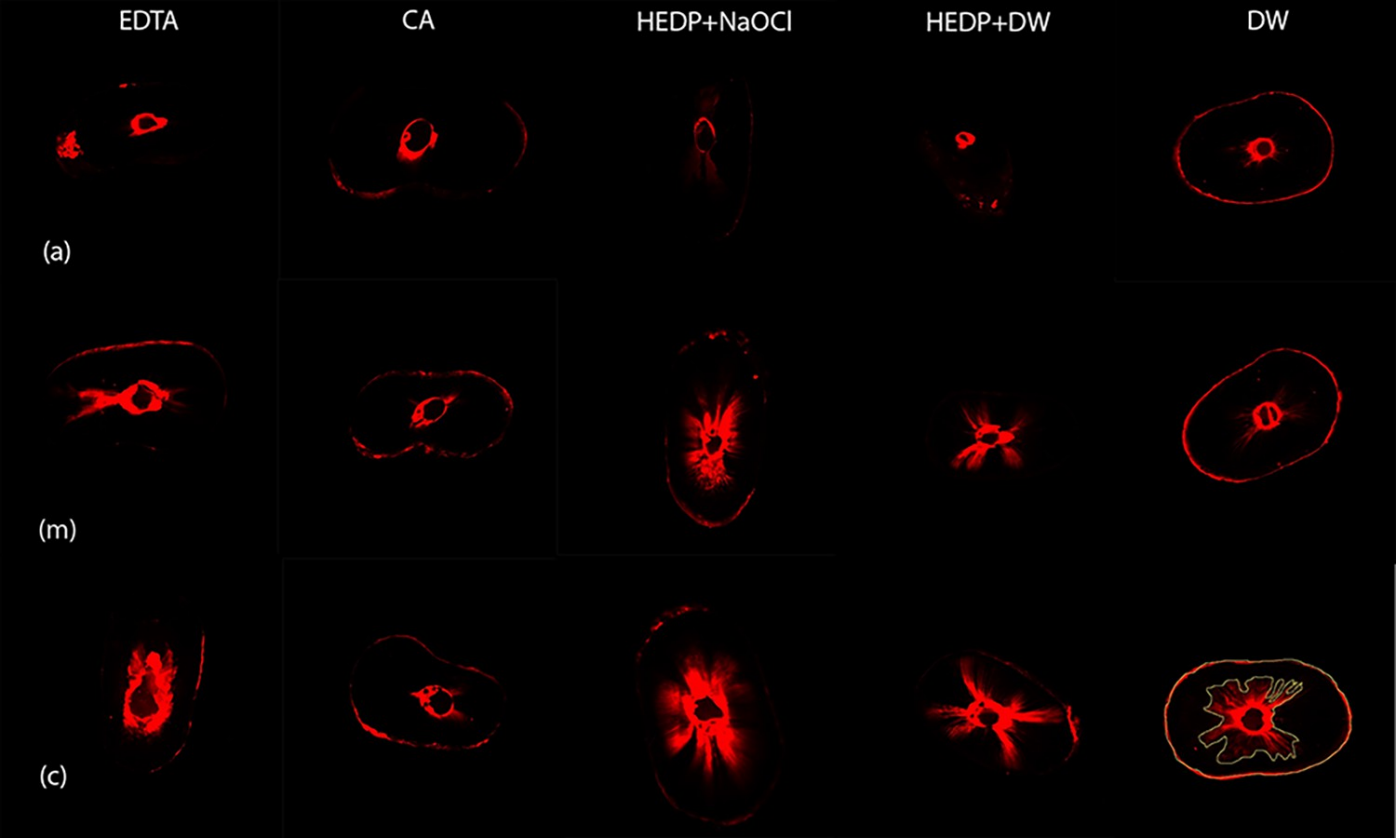
Confocal microscopy images of representative samples treated from each chelating solution group (columns) at each sectional level (rows). (×10 magnification, 7×7 tile scan mode). Columns in respective order: (1) EDTA, (2) HEDP, (3) CA, (4) DW. Rows represent the (a) apical, (b) middle and (c) coronal sections. An example of the calculation of area of sealer penetration in the outer wall is shown in 4(c). CA, citric acid; DW, distilled water; EDTA, ethylenediaminetetraacetic acid; HEDP, etidronic acid.

Fig 3 shows the representative images of each experimental group at each depth. The Kruskal–Wallis test revealed a significant difference on the basis of total penetration percentage (%) and the maximum penetration depth (mm). EDTA and CA presented the highest total penetration mean percentages (Table 3). With respect to the root canal depth, the values obtained with EDTA (18.14%) and CA (13.9%) were significantly higher than with HEDP + DW and DW (*p***<**0.001) in apical third. In the middle third, the highest values seen in EDTA, CA, and HEDP + NaOCl groups than those of the DW group (*p***<**0.001). At cervical level, EDTA, HEDP + NaOCl, and HEDP + DW groups showed significantly higher penetration than the DW group (*p* **=** 0.020). Comparison of root canal depth within test groups; sealer penetration was significantly higher in cervical than in apical for HEDP + NaOCl, HEDP + DW, and DW (*p***<**0.001). Supporting information of this study was made available [43].

**Table 3:** Mean total penetration percentage at each section level.

Percentage of penetration (%)		Apical	Middle	Cervical	*p*-value
**EDTA**	Mean±SD	18.14±16.61^Aa^	23.23±15.54^Aa^	34.61±17.28^Aa^	0.067
	Median (IQR)	11.36 (5.77–35.46)	18,85 (11.82–33.06)	37.36 (20.26–46.58)	
**CA**	Mean±SD	13.9±9.93^Aa^	19.51±12.56^Aa^	23.,26±15.8^ABa^	0.294
	Median (IQR)	11.25 (7.67–16.92)	15,22 (9.62–27.65)	16,24 (10.47–33.6)	
**HEDP + NaOCl**	Mean±SD	5.5±4^ABb^	14.6±2.64^Aa^	26.99±16.01^Aa^	<0.001*
	Median (IQR)	6.45 (1.22–7.91)	14.6 (13.36–15.98)	20.37 (17.73–37.23)	
**HEDP + DW**	Mean±SD	2.87±2.49^Bb^	8.94±8.5^ABb^	24.99±14.46^Aa^	<0.001*
	Median (IQR)	1.92 (0.78–5.22)	8.18 (3.18–11.1)	18.42 (14.12–35.32)	
**DW**	Mean±SD	2.27±1.81^Bb^	5.77±3.26^Ba^	10.38±7.34^Ba^	0.001*
	Median (IQR)	2.07 (0.55–3.74)	5.54 (2.62–8.6)	8.38 (7.28–10.91)	
	***p*-value**	<0.001*	<0.001*	0.002*	

The values are presented as means±SD or medians (IQR).

*Kruskal–Wallis test (*p*<0.05). Uppercase superscript letters indicate statistical differences between columns (A-B). Lowercase superscript letters indicate statistical differences between rows (a-b). Dunn–Bonferroni post-hoc test (*p*<0.05).

CA, citric acid; DW, distilled water; EDTA, ethylenediaminetetraacetic acid; HEDP, etidronic acid; IQR, interquartile range; SD, standard deviation

A significant difference in the maximum penetration depth at the sectional level was observed only between the HEDP + NaOCl and HEDP + DW groups (Table 4). At the apical level, EDTA (2.91 mm) and CA (2.57 mm) showed significantly higher values than DW (1.28 mm), whereas at the cervical level, a significant difference was observed between EDTA (4.03 mm) and DW (2.21 mm).

**Table 4:** Maximum depth of sealer penetration at each section level.

Percentage of penetration (%)		Apical	Middle	Cervical	*p*-value
**EDTA**	Mean±SD	2.91±1.4^Aa^	3.55±1.13^Aa^	4.03±0.64^Aa^	0.082
	Median (IQR)	2.87 (1.77–3.73)	3.49 (2.86–4.49)	4.21 (3.54–4.49)	
**CA**	Mean±SD	2.57±0.69^Aa^	2.8±0.96^Aa^	2.85±0.87^ABa^	0.017*
	Median (IQR)	2.88 (1.77–3.05)	2.68 (2.06–3.38)	2.74 (2.41–3.38)	
**HEDP + NaOCl**	Mean±SD	1.82±1.04^ABb^	2.72±1.29^Aab^	3.59±1.13^ABa^	0.879
	Median (IQR)	1.96 (0.58–2.4)	2.49 (1.65–3.78)	3.44 (2.55–4.55)	
**HEDP + DW**	Mean±SD	1.57±0.83^ABb^	2.86±0.95^Aa^	3.25±1.14^ABa^	0.002*
	Median (IQR)	1.48 (1.04–2.41)	2.99 (2.76–3.37)	3.15 (2.17–4.43)	
**DW**	Mean±SD	1.28±1^Ba^	2.36±1.11^Aa^	2.21±1^Ba^	0.054
	Median (IQR)	0.97 (0.51–1.72)	2.43 (2.03–3.17)	2 (1.47–2.58)	
	***p*-value**	0.007*	0.265	0.003*	

The values are presented as means±SD or medians (IQR).

*Kruskal–Wallis test (*p*<0.05). Superscript letters indicate statistical differences between columns. Lowercase letters indicate statistical differences between rows. Dunn’s Bonferroni post-hoc test (*p*<0.05).

CA, citric acid; DW, distilled water; EDTA, ethylenediaminetetraacetic acid; HEDP, etidronic acid; IQR, interquartile range; SD, standard deviation

## Discussion

The mechanical action of endodontic instruments used in root canal shaping results in the formation of a smear layer formation [7, 44]. Root-canal irrigation requires a chelating solution to remove this layer. Removing the smear layer enables penetration of the root canal sealer into the dentinal tubules, preventing reinfection [3] and enhancing sealer retention [32]. After root canal shaping, alternative agents with different characteristics are increasingly used in final irrigation regimens [33]. To our knowledge, this is the first study to evaluate the effects of chelating agents, including 9% HEDP mixed in two different forms, on smear layer removal and tubular sealer penetration, and to compare them with the effects of other widely used agents.

The effects of an HEDP solution on dentin depends on its concentration [45], similar to other common irrigation solutions [38]. EDTA and CA were used at concentrations of 10–17% and 10–50%, respectively [11]. The effectiveness of 17% EDTA and 10% CA for smear layer removal [13] and sealer penetration [38, 46] is similar, which is consistent with our findings. In our study, 9% HEDP + NaOCl presented a lower penetration percentage and depth than 17% EDTA and 10% CA, and higher values than 9% HEDP + DW. Therefore, the null hypothesis was rejected. This result is consistent with that of De-Deus et al., who reported that the demineralization kinetics of 9% HEDP were slower than those of 17% EDTA over time [47]. This might explain the lower penetration value of 9% HEDP+ DW observed in this study. However, previous studies have shown better effectiveness with 9% HEDP + NaOCl than with EDTA [36, 37]. Gawdat et al. [37] reported higher sealer penetration values for 9% HEDP + NaOCl than for NaOCl alone, followed by EDTA. These results contradict our findings, which can be attributed to the elimination of the organic portion of the smear layer upon the application of 9% HEDP.

Furthermore, 7% HEDP has a weaker calcium-chelating capacity than 17% EDTA and 10% CA [11], whereas 18% HEDP was found to remove similar but lower levels of calcium from dentin than 15% EDTA and 10% CA [48]. Moreover, the solution concentration affected the mechanical strength of the root dentin. It has been suggested that 17% EDTA and 19% CA, although adequate for complete removal of the smear layer, can affect the mechanical durability of dentin because of their high acidity [49]. A previous study showed no difference in the reduction of dentin microhardness between these two solutions [50]. Effective smear layer removal may require the prolonged use of a chelating agent [13], As 17% EDTA and 10% CA reduce dentin microhardness, a less aggressive alternative, such as 9% HEDP, may be suitable [11]. Further research is needed to assess the effect of prolonged use of a chelating agent on effective smear layer removal, sealer penetration, and dentin microhardness.

Even though there is a wide range of use of EDTA, citric acid or similar strong acid cleansers as final irrigant, there is still no precise protocol established among practitioners. Nevertheless, the irrigation regimen includes a chelating solution following root canal shaping because NaOCl is unable to remove inorganic debris or the smear layer formed after instrumentation. Hence, the recommended protocol uses EDTA or CA (strong chelators) after NaOCl treatment [5] as final irrigant. Therefore this study followed an irrigation similar protocol where every test group received a final irrigation with a chelating agent. The time required to use the final irrigant was another controversial [38, 51] and an influencing factor of the irrigation regimen. This study used a standardized 5 mL final irrigation protocol for 1 min similar to previously conducted studies [52].

Several agitation methods have been developed for effective root-canal debridement, including the use of root-canal brushes, ultrasonic devices, and sonic activation [7]. Although ultrasonic methods result in increased debris removal [22, 53], we used EndoActivator (Dentsply), which is a sonic activation system, primarily because its efficacy is comparable to that of ultrasonic irrigation [54] and its disposable polymer tip does not cut the dentin, whereas “it is difficult to control the cutting of dentin and, hence, the shape of the prepared root canal” with ultrasonic systems [7]. The irrigation solution was activated for 30 s according to Sabins et al. [53], who reported no significant differences in debris removal over longer periods.

SEM analysis revealed that smear layer removal improved with the use of 17% EDTA compared with the use of other agents. It was also highest in the coronal third, followed by the middle and apical thirds. These findings are supported by previous reports [38, 55, 56] and can be attributed to the lower tubular density in the apical region of the root canal. However, Kfir et al. [36] compared the same concentrations of HEDP and EDTA, and used the same debris removal scoring system as the SEM images used in our study. The authors did not report a difference between the two solutions, which may suggest an effect of solution interaction, as they prepared 9% HEDP with 3% NaOCl and did not perform interim saline irrigation between NaOCl and EDTA. Although SEM is an effective tool for evaluating smear layer removal from root dentin, it has several drawbacks such as subjective assessment and a reduced examination area [13]. To overcome these limitations, images were selected by an examiner blinded to the study protocol, calibrated prior to image analysis, and obtained under two magnifications for a more elaborate measurement of each specimen.

The smear layer can extend 40 μm into the dentinal tubules [5]. Therefore, and efficient removal of the smear layer will benefit the penetration of the obturation method into dentinal tubules and will impact the sealing of the root canal space. The amount of smear layer removed was calculated using SEM; however, the number of voids filled by the sealer requires additional measurement because it is affected by various parameters, such as dentin wettability, surface tension, and viscosity of the sealer [57]. Therefore, calculating the maximum penetration depth is beneficial for obtaining information on the longest distance penetrated by a sealer. However, this is insufficient to completely represent tubular sealer penetration, because this penetration is dispersed along the dentinal area. Hence, the maximum penetration percentage was calculated based on the previous studies [58, 59].

We evaluated the penetration depth and percentage of tubular sealers. Sealer penetration into the dentinal tubules inactivates bacteria, prevents regrowth [3], and increases penetration [46]. Light microscopy, SEM, and CLSM have been used to visualize root canal sealers in the dentinal tubules. CLSM can regulate the field depth to assemble many optical sections, even thick specimens, and reduce the background information from the focal plane [27]. Tedesco et al. found that CLSM forms high-contrast points, allowing for better visualization at various depths. CLSM improves intratubular sealer penetration visualization of sealer tag depth and quantity. Tubular sealer penetration may exceed CLSM image visibility [56]. A tile-scan setting is used to overcome this limitation.

CLSM sealers must be labeled with fluorescent dyes for visibility. Rhodamine B (Sigma-Aldrich, St. Louis, MO, USA) was used to identify sealers [58] without affecting their physical properties [60]. A recent study found that leakage of the sealer mixture passively diffuses rhodamine into the dentinal tubules, which could overestimate the results [61]. Further studies evaluating sealer penetration should use more stable dyes mixed with root canal sealers to overcome this limitation. In this regard, the use of a sealer that diffuses into the tubules equally and shows adequate attachment properties would also be beneficial for the observing purposes. In this study a resin -based sealer, AH Plus, was chosen during study intervention due to its adequate properties such as viscosity, radiopacity, and easy handling. However, the rising trend of bioceramic -based sealers in clinical practice is noticeable in the last few years [62]. A recent study has shown a higher tubular sealer penetration in calcium-silicate cements in comparison to AH Plus [63] Even though that the use of bioceramics are a rising trend in endodontic clinical practice due to their increased biocompatibility and chemical attachment to the dentinal wall, AH plus, a resin-based sealer, remains to be considered as the gold-standard.

According to our findings, the canal depth had significant influence on the tubular sealer penetration. Coronal sections showed higher penetration and maximal sealer depth than apical sections. These findings were consistent with the increased removal of the smear layer from the coronal part of the root canals. Several studies comparing the penetration abilities of various root canal sealers using SEM [55] and CLSM [64] have demonstrated better penetration at the coronal level [40]. Complete preparation of the apical portion remains a major clinical challenge because of canal deviations or deltas primarily located in this part of the root canal. Although these drawbacks have been addressed through apical enlargement and delivery of the root canal sealer using a high-speed instrument, low penetration levels in the apical part persist, which may be attributed to decreased dentin permeability owing to the lower density [60, 61] and irregular direction of the tubules [65].

This study had some limitations. First, the preparation of the HEDP solutions differed from that used in most studies. The use of HEDP with NaOCl as a continuous irrigation agent during endodontic treatment is well established [20, 40]. However, it can also be mixed with distilled water in clinical settings [21]. To solely examine the efficacy of HEDP on smear layer removal, in addition to its bacterial elimination properties, a test group containing 9% HEDP as a single final irrigant mixed with distilled water was also added. Second, the ability of dye diffusion into dentinal tissues also delivered another limitation. De-Deus et al. has previously mentioned that the zones in CLSM scans not presenting sealer indicates sclerotic dentine. Even though the effect of this phenomenon was tried to be lowered during sampling, it is possible to occur especially at the apical part of the root canal [23]. However, it is also possible that due to the limited age range of the included age of the patient population and the fact that only extracted single-rooted lower premolars were included in the study, the diversity of the study could be hindered and may not represent a complex clinical environment. Future studies with a comparable in-vitro nature that includes a wide range of sample population would contribute in resembling the clinical impact. Lastly, limitations related to smear layer evaluation were present. The use of SEM to evaluate the dentinal walls, as previously described [36, 40]. However, this method has limitations in terms of its reproducibility and validity. De-Deus et al. [23] previously criticized the use of SEM for the evaluation of smear layer removal because of the lack of standardization. Moreover, it is impossible to achieve an entire view of the canal using this method because it only allows the assessment of limited areas of the canal wall. Therefore, future studies utilizing advanced evaluation methods, such as micro-computed tomography, may provide a better understanding. Nevertheless, the use of the study methods by a trained technician and the calibration of observers helped minimize discrepancies related to the standardization and reproducibility of the methods, such as those related to the area selection of the samples.

In the light of our findings, the use of strong chelating agents as EDTA and citric acid presented improved outcomes in terms of sealer penetration and smear layer removal whereas continuous or single irrigation with a HEDP agent did not bring any significant improvements in terms of sealer penetration. However, the time-effective nature of continuous irrigation with less degrading effect of a mild chelating agent can also benefit for the prognose of the treatment. Further long-term clinical studies or comparative analyses considering the impact of chosen chelating agent on the prognose of the treatment would be a valuable contribution to the existing literature.

## Conclusions

Within the limitations of this study, the use 17% EDTA and 10% CA showed better efficiency in tubular sealer penetration and smear layer removal above all other tested agents. With respect to root canal depth, maximum sealer penetration and smear layer removal was observed in the coronal third. The findings of this study suggests that the use of a strong chelating agent as final irrigant together with the solution well-diffused inside the root canal may have a significant contribution to the efficient sealing of the root canal.
